# Evaluation of drive-through pharmacy service in Queen Elizabeth Hospital Malaysia

**DOI:** 10.1186/s40545-020-00221-7

**Published:** 2020-06-11

**Authors:** Jerry Ee Siung Liew, Aizan Adifarhan bin Abdul Gapar, Lik Ting Shim

**Affiliations:** 1 Pharmacy Department, Queen Elizabeth Hospital, Locked bag: 2029, 88586 Kota Kinabalu, Sabah Malaysia; 2 Pharmacy Department, Queen Elizabeth Hospital, Locked bag: 2029, 88586 Kota Kinabalu, Sabah Malaysia; 3 Pharmacy Department, Queen Elizabeth Hospital, Locked bag: 2029, 88586 Kota Kinabalu, Sabah Malaysia

**Keywords:** Hospital pharmacy services, Malaysia, Value-added service, Drive-through pharmacy service, Personal satisfaction

## Abstract

**Background:**

In 2015, the drive-through pharmacy was first introduced in Queen Elizabeth Hospital (QEH), Malaysia as one of the pharmacy value-added services. Therefore, it is imperative to review the service for further amelioration to fulfil patients’ needs and expectations.

**Objective:**

The aim of this study is to evaluate the drive-through pharmacy service in Queen Elizabeth Hospital, Malaysia.

**Methods:**

A cross-sectional observational study was conducted from July to December 2018. The questionnaire was developed and underwent thorough validation process which yielded a Cronbach’s alpha reliability score of 0.9130. Satisfaction was calculated by mean percentage score (0% (dissatisfied) to 100% (satisfied). All data were analysed descriptively and thematic analysis was used in analysing open-ended question.

**Results:**

Compliance in obtaining medication was at 96.3% with a given two-week grace collection period. Insufficient quantity of medications (33.3%) was the highest near-missed medication errors occurred at the drive-through pharmacy. The mean satisfaction percentage score for all patients were 76.6% ± 8.1. A total of 69.2% (*n* = 83) were “very satisfied” while 30.8% (*n* = 37) were “satisfied” with the service. Among the reasons for satisfaction are convenience in getting medication refills (*n* = 74, 62%), short waiting time (*n* = 75, 63%) and knowledgeable dispensers (*n* = 87, 73%). A handful of patients were “dissatisfied” with the opening hours (*n* = 14, 11.7%) and the location of the drive-through pharmacy service (*n* = 19, 15.8%).

**Conclusion:**

Compliance in medication collection is acceptable within stipulated grace period. Despite low occurrence, identification of near-missed medication errors provides useful insights for future improvement of the service. Generally, our patients are satisfied with the service. However, we need to re-evaluate on the opening hours and location of the service.

## Introduction

In line with the transforming and evolving health care landscape, there is a need for pharmacists to take on more active roles in extending pharmacy services. Various methods have been proposed in other countries to reduce patient waiting time. This includes automated dispensing system, manpower scheduling plan, electronically delivered prescription and redesign of pharmacy workflow [1, 2].

In 2003, the concept of Pharmacy Value Added Services (VAS) was introduced by the Malaysian Pharmaceutical Services Division, Ministry of Health Malaysia. VAS is an innovative dispensing system which provides patients with alternative means to collect their monthly drug supply from the hospital. Among the services offered in VAS in Malaysia includes Integrated Drug Dispensing System, Postal Medicine, Drive-through Pharmacy, and SMS and Take. Despite the convenience and advantages of VAS, patients’ adoption rate is still low because the services are still a new concept for most patients [3].

The first drive-through pharmacy in Malaysia started in Pulau Pinang in 2008. Following the success of the project, the drive-through pharmacy was made as one of the key performance indicators (KPI) for the Minister of Health in 2010. In 2015, the Drive-through Pharmacy was introduced in Queen Elizabeth Hospital, Kota Kinabalu. According to the VAS registration statistics, about 1000 prescriptions were dispensed through the drive-through pharmacy in our hospital per month.

Despite the positive feedback from literatures, we did not find any specific literature that reported the performance and service satisfaction of drive-through pharmacies in East Malaysia setting. Evaluation of patients’ satisfaction is important as it has been used as one of the key indicators of service quality and delivery in the Malaysia healthcare system [4]. Furthermore, the results obtained from this study will help in rectifying identified weaknesses as well as strengthening the drive-through pharmacy service based on feedback and comments.

### Aim of the study

Hence, the main objective of this study is to evaluate the drive-through pharmacy service in Queen Elizabeth Hospital. In conducting this study, we have focused on 3 main domains; Compliance rate of obtaining medication refills at prefixed appointment, occurrence of near-missed medication errors at the drive-through pharmacy and patients’ level of satisfaction on the service provided.

## Methods

### Study settings

A cross-sectional observational study was carried out at the drive-through Pharmacy Service, Queen Elizabeth Hospital for 5 months (July until December 2018). On average, this service processes approximately 1000 prescriptions in a month. Drive-through Pharmacy enables patient to collect their monthly supply of medications without having to be present at the conventional pharmacy counter. Patients who subscribed for this service will drive their vehicles up to a designated area located within the hospital compound and their medications will be dispensed to them from a window. This area was easily accessible to cars and patients generally did not need to leave their car. During peak hours, a line of car is created and progressed in a single direction towards drive-through area. This service opened during weekdays, from 8 am to 5 pm.

### Definition of terms used

Several important terms used in this study defined as below (Table 1).

**Table 1:** Definition of Terms

	TERMS	DEFINITION
1.	Compliance	Patients are compliant towards medication collection appointment at the drive-through pharmacy. Medication collection appointment was decided between patients and pharmacist after previous supply. A 2-week extension period is allocated if patients missed initial appointment.
2	Patients	Referring to patients or caregivers that come to drive-through pharmacy to collect their medications.
3	Repeat prescriptions	All drug prescriptions which require patient to come back for additional drug supply. Prescribed prescriptions must be of duration more than 1 month.
4	Pharmacy personnel	Referred to either pharmacist or assistant pharmacist on duty at the drive-through pharmacy.

### Study population

Our research aimed to generalize the results towards two study population. First study population was prescriptions that were received daily at the drive-through pharmacy. This included both new and pre-existing prescriptions. We aimed to investigate patients’ compliance in obtaining medication refill during pre-fixed appointment date and types of near-missed medication errors that occurred.

Second study population was patients that came to Drive-through Pharmacy Service to obtain their medication. Patients’ level of satisfaction in using this service was evaluated.

### Inclusion and exclusion criteria

In order to ensure homogenous study samples that address specific study populations, the inclusion criteria were all prescriptions registered with Drive-through Pharmacy Service and patients or caregivers who have utilised this service.

Study subjects would be excluded if patient/caregivers came to collect their medication out of designated working hours, patient or caregiver with language barrier and who refused to cooperate. Certain criteria were imposed before a prescription was eligible to use this service. Medication collection at the drive-through pharmacy must be during office hours (Monday to Friday, 8 am to 5 pm), prescriptions that were deemed not suitable for this service include prescriptions that included dangerous or psychotropic drugs, short expiry drugs, drugs supply that were restricted due to stock issue, drugs that require pharmacist’s counselling, one-off prescriptions and repeat prescriptions with less than 1 month duration.

### Data collection method

Prescriptions presented at the drive-through pharmacy were screened daily for eligibility throughout study period. While preparation was made to dispense medication, study investigators would record parameters of interest such as number of patients, number of prescriptions dispensed, number of items per prescriptions, compliance percentage, number of defaulters and types of near-missed medication error that occurred. At the same time, patients contact details were recorded. A special time was allocated to contact patients via telephone. This is to prevent an increase in waiting time at drive-through pharmacy if data were recorded all at the same time. During phone interviews, a Patient Information Sheet was used to brief patients about the study. Patients had the complete discretion either to participate or to decline to join the study. If patients agreed in joining the study, verbal consent was obtained. Subsequently, study investigators would proceed with a guided interview using a validated questionnaire to evaluate patients’ level of satisfaction on drive-through pharmacy services. In order to ensure data collected were precise and consistent, a data dictionary was prepared. Furthermore, all study investigators were briefed and trained in how to extract and record relevant information using prepared data collection form.

### Study endpoints and outcomes

Primary outcome of this study was compliance in obtaining medication refill during prefixed appointment at the drive-through pharmacy and types of near-missed medication errors that occurred throughout the study period. Secondary outcome was to measure patients or caregiver satisfaction in utilising our drive-through pharmacy service.

### Study instruments

Two study instruments were utilised in this study: A set of standardized data collection form that recorded all parameter of interest and a set of questionnaires (Additional File 1). This questionnaire was developed to assess patient’s level of satisfaction scale in using drive-through pharmacy service. We carried out a thorough validation process for the questionnaire to ensure that the data obtained were reproducible and accurate.

To ensure the appropriateness of the questionnaire content, the items developed in this questionnaire were jointly discussed by two senior pharmacists, pharmacist in charge of drive-through pharmacy and four junior pharmacists. This was to ensure the components developed were appropriate according to the underlying study concept. As a result, a pre-validated 3 component of Drive-through Pharmacy Service Satisfaction questionnaire was developed. First component was basic demographic data such as age, gender, distance from home to the drive-through Pharmacy, patients or caregivers, occupation, level of education, drive-through pharmacy experience, duration using the service and methods in knowing about the service. Second component was 15 items scales that were purported to measure Drive-through Pharmacy Satisfaction Scale. The last component was 3 items open-ended questions (Question 5,6 and 7). The reason open-ended questions were included is because these questions require patients to prioritise issues based on their experience. Furthermore, open-ended questions may uncover hidden problems that we might have missed. Apart from that, we also incorporated 3 additional questions on overall service satisfaction (Question 4), whether they would continue to use the service (0–100%) (Question 8) and whether they would recommend the service to others (0–100%) (Question 9). All the items were pre-tested on 5 patients that did not participate in the final data collection. Pre-testing of questionnaires was carried out until there were no more issues pertaining to understanding and interpretation of the questionnaire.

Item homogeneity was assessed by item-partial total correlation and Cronbach’s Alpha coefficient. The construct validity of the 15 items component to measure Drive-through Pharmacy Satisfaction Scale was assessed by exploratory factor analysis. Alpha coefficients for Drive-through Pharmacy Satisfaction Scale was 0.9130. There were 3 factors with Eigenvalue more than one. However, noted that 15 items load highly on one factor only (Table 2), which indicates there was only one important construct for the scale. Therefore, we decided not to separate the items into categories (Tables 3).

**Table 2:** Factor Analysis of Drive-through Pharmacy Satisfaction Scale (Principal Factor Method)

FACTOR	EIGENVALUE
Factor 1	6.94110
Factor 2	0.75184
Factor 3	0.61548
Factor 4	0.38257
Factor 5	0.23920
Factor 6	0.13226
Factor 7	0.11017
Factor 8	0.01828
Factor 9	−0.01987
Factor 10	− 0.05300
Factor 11	−0.09397
Factor 12	−0.13757
Factor 13	−0.14425
Factor 14	−0.17466
Factor 15	−0.24155

**Table 3:** Factor Loadings and Uniqueness of Drive-through Pharmacy Satisfaction Scale

VARIABLE	FACTOR1	UNIQUENESS
Q2_1	0.5629	0.6831
Q2_2	0.7297	0.4675
Q2_3	0.5072	0.7428
Q2_4	0.7024	0.5066
Q2_5	0.6620	0.5618
Q2_6	0.7197	0.4821
Q2_7	0.6366	0.5948
Q2_8	0.7236	0.4763
Q2_9	0.3758	0.8588
Q3_0	0.7826	0.3875
Q3_1	0.6246	0.6099
Q3_2	0.8050	0.3520
Q3_3	0.7823	0.3880
Q3_4	0.7646	0.4154
Q3_5	0.6838	0.5324

Each response from Drive-through Pharmacy Satisfaction Scale were given a score. Total score obtained were divided by the total score (possible maximum total score) times 1 hundred in order to obtain percentage of score for each patient. The percentage score ranges from 0 to 100%, with higher percentage score represent better satisfactory level of Drive-through Pharmacy Service.

### Ethics approval

This research was registered with National Medical Research Registry (NMRR) and it was approved by the Malaysian Institutional Review Board (IRB)/ Independent Ethics Committee (IEC) with NMRR ID of NMRR-18-1638-42,105 (IIR).

### Statistical analysis method

Data were analysed using STATA SE/12 (StataCorp LLC, College Station, TX, USA). Data were initially recorded in a prepared Microsoft Excel Spreadsheet before further analysis. Initial data cleaning was carried out to eliminate data entry error and to identify if there was any missing data. Descriptive statistic was employed as the main statistical analysis method. All normally distributed data was presented as mean and standard deviation (SD) whereas non-normally distributed data, they were presented as median and inter-quartile range (IQR). Categorical data was presented as frequency and percentage. Thematic analysis using qualitative approach was used in analysing open-ended question (Question 5,6 and 7). All interview responses were listed and grouped until saturation of themes was reached.

Separate sample size was calculated for both outcomes. For the first outcome which is compliance in obtaining medication refill during prefixed appointment, sample size was estimated based on 1-month pilot study. To detect a proportion of 95% compliance in obtaining medication refill during prefixed appointment at 80% power with precision of 5% and 0.05 alpha value, 92 days were required to be evaluated. In our study, we evaluated a total of 180 days which is more than the minimum required sample size.

Similarly, the second outcome which is the Drive-through Pharmacy Satisfaction Scale sample size was calculated based on pilot study. A total of 40 patients were recruited in the pilot study. To obtain a mean percentage score with standard deviation of 4.7 at 80% power with precision of 1% and alpha 0.05, a total of 107 patients were required. A total of 120 patients were evaluated in this study.

## Results

### Compliance of medication collection

A total of 2792 patients with at least one prescription per person (Total=3248 prescriptions) were analysed. Nine hundred and five patients (31.8%) came to collect their subsequent medication supply on the exact date given to them. However, majority of them (96.3%, 2678 patients) came to obtain medication supply within 2 weeks from the exact date given to them. There were 114 patients (3.7%) defaulted in in collecting subsequent medication supply. One hundred fourteen patients with 122 prescriptions (3.4%) and 382 (3.1%) were left uncollected.

### Occurrence of near-missed medication errors

The mean number of prescriptions dispensed per day was 39.5 ± 10.7. Out of all the prescriptions that had been dispensed, insufficient medication supplied accounted for the most reported near-missed medication error (33.3%, *n* = 8) while the least occurred near-missed medication error was medication supplied to the wrong patient (4.2%, *n* = 1) [Table 4].

**Table 4:** Near-Missed Medication Error Evaluation

TYPES OF NEAR-MISSED MEDICATION ERRORS	FREQUENCY (N)	PERCENTAGE (%)
Insufficient supply of drugs	8	33.3
Wrong drugs supplied	4	16.7
Poly-pharmacy	4	16.7
Missing drugs	4	16.7
Wrong label	3	12.4
Wrong Patient	1	4.2

### Level of satisfaction evaluation

#### Patients demographic

A total of 120 patients were recruited to answer the drive-through satisfaction questionnaire. Their age ranged from 18 to 70 years old with mean (SD) of 52.3 (15.3). The ratio of patient to caregiver among the patients is 7:3. Study population was predominantly females (54.2%, *n* = 65) and the mean distance from their homes to the hospital is 8.1 km. Majority of patients received tertiary level education (50.8%, *n* = 61) and are employed (52.5%, *n* = 63). A great percentage of patients (90%, n = 108) are existing users of the service with mean (SD) period of time (months) using drive-through pharmacy service of 18.3 (13.1). [Table 5].

**Table 5:** Baseline demographic

VARIABLE	N (%)
Age, mean (SD)	52.3 (15.3)
Gender	
Male	55 (45.8)
Female	65 (54.2)
Location (km), Mean (SD)	8.1 (10)
Collection	
Patient	76 (63.3)
Caregiver	44 (36.6)
Occupation	
Employed	63 (52.5)
Unemployed	57 (47.5)
Education	
Primary	4 (3.3)
Secondary	55 (45.8)
Tertiary	61 (50.8)
Period of time using drive-through pharmacy service (months), Mean (SD)	18.3 (13.1)

Overall, (69.2%, *n* = 83) were “very satisfied” while (30.8%, *n* = 37) were “satisfied” with the drive-through pharmacy services. Among the reasons that lead to high level of satisfaction include convenience in getting medications, great interaction between dispenser and patients, short waiting time and rectification of problems in a timely manner.

Opening hours and location of the drive-through pharmacy accounted for a notable percentage of dissatisfaction (11.7%, *n* = 14 and 15.8%, *n* = 19) respectively. However, the patients gave high rating in which they will continue to subscribe to the service in the future (95.4 ± 7.1) and would be recommending others to use the service (94 ± 8.8). The mean score for all subjects were 76.6% ± 8.1 (Table 6).

**Table 6:** Mean score of drive-through satisfaction scale

PHARMACY DRIVE-THROUGH SERVICE	RESPONSES, N	%
Opening hours (Office hours 8–5 pm, Monday to Friday)		
Dissatisfied	14	11.67
Satisfied	51	42.5
Very Satisfied	55	45.8
Interaction between dispenser and patients/caregiver		
Satisfied	33	27.5
Very Satisfied	87	72.5
Location		
Dissatisfied	19	15.8
Satisfied	42	35
Very satisfied	59	49.2
Convenience in getting medication		
Dissatisfied	8	6.7
Satisfied	38	31.7
Very satisfied	74	61.7
Rectification of problems in timely manner		
Dissatisfied	5	4.2
Satisfied	37	30.8
Very satisfied	78	65
Short waiting time.		
Dissatisfied	1	0.8
Satisfied	44	36.7
Very satisfied	75	62.5
Clear direction		
Very Dissatisfied	3	2.5
Dissatisfied	9	7.5
Satisfied	56	46.7
Very satisfied	52	43.3
Quantity and quality of medication dispensed		
Very Dissatisfied	1	0.8
Dissatisfied	4	3.3
Satisfied	33	27.5
Very satisfied	82	68.3
Overall Service Satisfactory Level		
Satisfied	37	30.8
Very satisfied	83	69.2
Would you continue to use this service? (Mean SD)		95.4 (7.1)
Would you recommend this service to others? (Mean SD)		94 (8.8)
Percentage of score, Mean (SD)		76.6 (8.1)

Subjective questions with regards to the drive through pharmacy service were also incorporated in order to obtain more accurate responses. Saturation of themes was achieved after the survey questionnaires were answered. The subjective response from drive through pharmacy users in terms of quality and improvements were outlined in Table 7.

**Table 7:** Subjective response from drive through pharmacy users on the overall performance of the service

THEME/RESPONSE	COMMENT	QUOTE
Excellent / Service did well		
1. Convenience (less hassle)	Many drive-through pharmacy users agree that the process of obtaining medication is less time consuming than the conventional pharmacy counter collection.	I do not have to queue up to take a number and wait for my number to be called before getting my medications. (Respondent 1, male)
	A large number of patients favour this service because they do not have to find parking.	… no parking lots in the hospital. Plus, I have a busy schedule and this service saved me a lot of time. (Respondent 2, female)
2. Knowledgeable personnel	Well-trained and client-friendly dispensers are among the positive feedback received by drive through pharmacy users.	Dispenser is polite, helpful and able to answer questions asked. (Respondent 3, Male)
Dislikes about the service		
1. Accessibility / Location	Many users expressed dissatisfaction that the drive through pharmacy is not conveniently accessible due to narrow roads.	… limited space for cars to pass through, if a lot of cars will create traffic jam. (Respondent 4, Male)
	Location of the drive through pharmacy is not strategic since it is located behind hospital canteen.	… hard to locate the drive through pharmacy for first timers & lack of signage (Respondent 5, Female)
2. Operating Hours	A number of drive through pharmacy users does not agree that the operating hours which is only during weekdays (8 am to 5 pm)	I am working during weekdays, it is hard for me to go out to collect medications at the drive through pharmacy. (Respondent 6, Female)
3. Service restrictions	Registrations for the service is not allowed for prescriptions containing low stock items (Controlled quantity of drugs due to insufficient stock)	....Limiting the service will bring inconveniences to the users as they will need to spend more time to get their medications, (Respondent 7, Male)

## Discussion

The compliance in obtaining medication on the date of collection and the occurrence of near-missed medication errors were evaluated in this study. Out of 2792 patients included in the analysis, 31.8% of the patient (n = 905) came on the exact date of refill given and 96.3% of them (n=2678) came within grace period of 2 weeks of the refill date. During this period, medication packages are still well kept according to recommended medication storage condition. There was only a handful of patients (3.7%, n = 114) completely defaulted the refill date. This indicates that in our settings, drive through pharmacy patients were more likely to be compliant to the collection dates when a 2-week grace period is introduced.

We also evaluated the occurrence of near-missed medication errors at the drive-through pharmacy. Previous findings had unveiled that pharmacists believed that drive-through pharmacy are subjected to extra processing steps that can contribute to risk of delays, lack of counterchecking and eventually medication errors [5]. Insufficient quantity of medication accounted for the most reported near-missed medication error followed by poly-pharmacy, wrong drugs supplied and wrong labelling. A reminder system via text message at least 3 days before pre-fixed appointment date should be implemented to minimize the risk of non-compliance. No linkages of computerized pharmacy system among other hospitals imposed a high risk of poly-pharmacy because most public hospitals in Malaysia are running on single-centred pharmacy information system. Dedicated pharmacists and adequate space for preparation and counterchecking of drive-through parcel medications are essential to reduce the incidence of near-missed medication errors in the long term. Despite low occurrence in our setting, identification of near-missed medication errors will help to provide useful insights for future improvement of the drive-through pharmacy service.

Very few researches have investigated on patient satisfaction and the factors that affect the quality of a drive-through pharmacy service. It was observed that a high number of our patients (69.2%, *n* = 83) were “very satisfied” with the service particularly due to convenience in collecting refill medications, short waiting time, timely rectification of problems and helpful personnel. This is also reflected in our subjective responses given by patients that agree drive-through pharmacy provides less hassle in terms of limited parking and high volume of patients at the hospital.

The high degree of satisfaction in our study are in line with a local study in Pulau Pinang which reported that patients were satisfied with the service as it managed to reduce waiting time (63.2%, *n* = 21), offer comfortable service hours (61.4%, *n* = 35) and situated at an accessible location (36.8%, *n* = 21) [6]. A study carried out in a tertiary hospital in Kelantan also demonstrated a high degree of satisfaction to the drive-through pharmacy service (61.2%, *n* = 237) and majority of patients perceive the service as useful to the public [7]. Among the VAS in Malaysia, drive-through pharmacy achieved higher satisfaction level than mail pharmacy service and call-and-collect service [8]. In Taiwan, a study on the first drive-through pharmacy in the country managed to provide patients with a convenient and quicker alternative compared to ordinary pharmacy collection [9].

However, factors such as operating hours and location of the drive-through pharmacy accounted for a notable percentage of service dissatisfaction (11.7%, *n* = 14 and 15.8%, *n* = 19) respectively. This is due to the fact that our drive-through pharmacy only operates on weekdays and not during weekends and after office hours. The drive-through pharmacy is also situated behind a hospital canteen building with narrow roads which could have limited the vehicles’ pathway.

We also managed to garner a few key areas for improvement in our study based on our subjective open-ended questions. Extending operating hours of the drive through pharmacy during after office hours and weekends is one of the main suggestions by patients. Majority of our patients are employed and with tertiary education background which limited them from coming to the drive-through pharmacy during normal working hours. Widening of the one-way narrow road is necessary to improve the accessibility to the drive-through pharmacy during peak hours or during unloading and loading process at a nearby canteen. Clear signage to the drive-through pharmacy also has been suggested by patients to provide accurate direction especially for first time users.

This study adds on to the current knowledge of drive-through pharmacy. In our study, majority of our patient are educated and prefer a faster and convenient alternative to collect monthly supply of medications. We also evaluated the compliance of patients in obtaining medication and the occurrence of near-missed medication errors which has not been done in previous studies. Furthermore, this study provides detailed breakdown of key areas of satisfaction, dissatisfaction and improvement in building a successful drive-through pharmacy.

Several limitations were detected in the study. Since it is a cross-sectional single centre study and the findings might not be representative of other settings with a bigger or smaller size of population. Furthermore, this study did not capture certain important factors which may be predictors of satisfaction including residence and severity of comorbidities of patients. Finally, this study is susceptible to recall bias and self-reflect bias.

## Conclusion

This study shows that compliance in medication collection is acceptable (96.3%) within stipulated grace period. Despite low occurrence, identification of near-missed medication errors provides useful insights for future improvement of the service. Generally, our patients are satisfied with the service. However, we need to re-evaluate on the opening hours and location of the service.

## Supplementary information

**Additional file 1.** Drive-through Pharmacy Service Satisfaction Questionnaire.

## Data Availability

Research data set may be obtained in writing with corresponding author.
