# Supplementary material for: Evaluation of drive-through pharmacy service in Queen Elizabeth Hospital Malaysia
Source: J Pharm Policy Pract. 2020 Jun 11;13:27. doi: 10.1186/s40545-020-00221-7 (PMC7288545; doi:10.1186/s40545-020-00221-7)
Supplement: Supplementary file 1 — Additional file 1. Drive-through Pharmacy Service Satisfaction Questionnaire. [file 40545_2020_221_MOESM1_ESM.docx]

**DRIVE-THROUGH PHARMACY SATISFACTION SCALE**

**SECTION A: Patient Demographic (Please Fill in ALL data, No MISSING data)**

1. Age: _______ (in 2018)

1.2. Gender: □ Male

□ Female

1.3. Location of Resident: __________________ (Exact Place, Please be SPECIFIC)

1.4. Collecting data: □ Patient

□ Caregiver

1.5. Occupation : □ Unemployed

□ Employed

1.6. Education: □ Primary

□ Secondary

□ Tertiary

1.7. History of using Drive Through: □ First Time User

□ Existing User

1.8. How do you know about Drive Through Service: □ Family Members/Relatives

□ Friends

□ Pamphlets/Advertisement

□ Social Media (Newspaper, radio)

□ Health Care Providers

1.9. How long have you been using Drive Through: _____ (Months) **OR** ____(Years)

**Section B: Drive-through Pharmacy Service Satisfaction (Question 2 to 4)**

Please rate according to scale stated below:

| **Very Dissatisfied** | **Dissatisfied** | **Satisfied** | **Very Satisfied** |
| --- | --- | --- | --- |
| **1** | **2** | **3** | **4** |

**2. Drive-through Pharmacy Service Evaluation**

| 2.1 | Opening hours (Office hours 8-5 pm, Monday to Friday) |  |
| --- | --- | --- |
| 2.2 | Interaction between dispenser and patients/caregiver |  |
| 2.3 | Location |  |
| 2.4 | Convenience and easy accessibility |  |
| 2.5 | Implication of weather on the service |  |
| 2.6 | Problem identification and solution in timely manner |  |
| 2.7 | Waiting time. |  |
| 2.8 | Dispenser are knowledgeable and helpful |  |
| 2.9 | Adequate direction and sign board. |  |
| 3.0 | Medication dispensed |  |
| 3.1 | Quantity of medication dispensed until next refill date |  |
| 3.2 | Drug label |  |
| 3.3 | Appropriate packaging |  |
| 3.4 | Flexibility in setting up appointment date |  |
| 3.5 | Procedure in obtaining medication after appointment date. |  |

**4. Overall satisfaction level of Drive-Through Pharmacy Service**

| Overall Service Satisfaction |  |
| --- | --- |

**5. What do you think we can improve our Drive-Through Pharmacy Service?**

**(*If no comment, please write “None”*)**

___________________________________________________________________________________________________________________________________________________________________________________________________________________________

**6. What do you think we did really well?**

**(*If no comment, please write “None”*)**

___________________________________________________________________________________________________________________________________________________________________________________________________________________________

**7. What do you dislike about Drive-Through Service?**

**(*If no comment, please write “None”*)**

___________________________________________________________________________________________________________________________________________________________________________________________________________________________

**8. Would you continue to use this service?**

0%

10%

20%

30%

40%

50%

60%

70%

80%

90%

100%

**9. Would you recommend this service to others?**

0%

10%

20%

30%

40%

50%

60%

70%

80%

90%

100%
